# An Inducible Fusaric Acid Tripartite Efflux Pump Contributes to the Fusaric Acid Resistance in *Stenotrophomonas maltophilia*

**DOI:** 10.1371/journal.pone.0051053

**Published:** 2012-12-07

**Authors:** Rouh-Mei Hu, Sih-Ting Liao, Chiang-Ching Huang, Yi-Wei Huang, Tsuey-Ching Yang

**Affiliations:** 1 Department of Biotechnology, Asia University, Taichung, Taiwan; 2 Department of Biotechnology and Laboratory Science in Medicine, National Yang-Ming University, Taipei, Taiwan; 3 Department of Medical Laboratory Science and Biotechnology, China Medical University, Taichung, Taiwan; 4 Graduate Institute of Microbiology and Public Health, National Chung-Hsing University, Taichung, Taiwan; Université Paris Descartes; INSERM, U1002., France

## Abstract

**Background:**

Fusaric acid (5-butylpicolinic acid), a mycotoxin, is noxious to some microorganisms. *Stenotrophomonas maltophilia* displays an intrinsic resistance to fusaric acid. This study aims to elucidate the mechanism responsible for the intrinsic fusaric acid resistance in *S. maltophilia*.

**Methodology:**

A putative fusaric acid resistance-involved regulon *fuaR*-*fuaABC* was identified by the survey of the whole genome sequence of *S. maltophilia* K279a. The *fuaABC* operon was verified by reverse transcriptase-PCR. The contribution of the *fuaABC* operon to the antimicrobial resistance was evaluated by comparing the antimicrobials susceptibility between the wild-type strain and *fuaABC* knock-out mutant. The regulatory role of *fuaR* in the expression of the *fuaABC* operon was assessed by promoter transcription fusion assay.

**Results:**

The *fuaABC* operon was inducibly expressed by fusaric acid and the inducibility was *fuaR* dependent. FuaR functioned as a repressor of the *fuaABC* operon in absence of a fusaric acid inducer and as an activator in its presence. Overexpression of the *fuaABC* operon contributed to the fusaric acid resistance.

**Significance:**

A novel tripartite fusaric acid efflux pump, FuaABC, was identified in this study. Distinct from the formally classification, the FuaABC may constitute a new type of subfamily of the tripartite efflux pump.

## Introduction

*Fusarium* is a large genus of filamentous fungi widely distributed in soil. The genus includes a number of economically important plant pathogenic species, such as *Fusarium graminearum* and *Fusarium oxysporum* f.sp. *cubense*. Fusaric acid (5-butylpicolinic acid), a mycotoxin produced by several *Fusarium* species [1], is firstly known to decrease plant cell variability [2], [3]. Fusaric acid is reported to be toxic to some microorganisms such as *Pseudomonas fluorescens* and mycobacteria [4], but is not generally used as an antimicrobial in clinic. Little is known about fusaric acid resistance in microorganisms. A fusaric acid-resistance associated operon has been reported from *Burkholderia cepacia* [5].

*Stenotrophomonas maltophilia*, an aerobic, nonfermentative, Gram-negative bacterium, is ubiquitous in nature, including soil, water, plants, and animals [6]. It is a member of endophytic bacteria, which can be isolated from plant rhizosphere, roots, and stems. *S. maltophilia* can generate antifungal organic compounds [7], plant growth factors [8], hydrolytic enzymes [9], and has been used for microorganism-based biological control in agriculture. Owing to the same habitats of endophytic *S. maltophilia* and *Fusarium*, the fusaric acid produced by *Fusarium* is a challenge for the survival of *S. maltophilia*.

In addition to its role in biocontrol, *S. maltophilia* is also an opportunistic human pathogen, causing nosocomial infection and community acquired [10]. Treatment of *S. maltophilia* infection is difficult since this pathogen is characterized by intrinsic and acquired resistance to a variety of antibiotics. The known mechanisms to combat the antimicrobial compounds in *S. maltophilia* include antibiotic hydrolysis or modification, target genes modification, membrane permeability alteration, and efflux pump overexpression [11]. Among them, the efflux pump is a crucial mechanism to remove a variety of toxic compounds and help bacteria to escape the attacks either from medical treatment or from natural environmental compounds. According to the structural characteristics, the efflux pumps are divided into five families: the resistance-nodulation-cell division (RND), the major facilitator superfamily (MFS), the small multidrug resistance (SMR), the multidrug and toxic compound extrusion (MATE), and the ATP-binding cassette (ABC) [12]–[14]. In Gram-negative bacteria, the RND, MFS, and ABC pumps may form a tripartite system to extrude the substance directly from cytoplasm to extracelluar environment. Tripartite pumps consist of an inner membrane protein (IMP), an outer membrane protein (OMP), and a membrane fusion protein (MFP) to link the IMP and the OMP. The genes encoding the IMP, OMP and MFP are generally organized into an operon. The known MDR pumps are generally chromosomally encoded, evolutionarily conserved, and may play a critical role in making bacteria to adapt the stresses occurring in their own habitats. With respective to the energetics of these efflux pumps, RND-, MFS-, SMR-, and MATE-type pumps use an electrochemical potential of protons across the cytoplasmic membrane as the energy source to extrude substrates. ATP hydrolysis is the energy source for the ATP-type pump to function [15].

A fusaric acid-resistance-involved tripartite FuaABC pump was identified in *S. maltophilia* in this study. The involvement of an AraC-type transcriptional regulator, FuaR, in its expression was investigated. Furthermore, the impact of the overexpressed FuaABC pump on antibiotic resistance was evaluated.

## Materials and Methods

### Bacterial Strains, Media, and Growth Conditions

Table 1 lists the bacterial strains, the plasmids, and the primers used in this study. All cultures were grown in LB broth at 37°C with shaking. PCR primer design was based on sequence data obtained from the *S. maltophilia* K279a genome [16].

**Table 1:** Bacterial strains, plasmids, and primers used in this study.

Strain or plasmid	Genotype or properties	Reference
*S. maltophilia*		
KJ	Wild type, a clinical isolate from Taiwan	[19]
KJFua23	A chromosomal *fuaABC-xylE* transcription fusion construct of *S. maltophilia* KJ	This study
KJΔFuaR	*S. maltophilia* KJ *fuaR* deletion mutant; *ΔfuaR*	This study
KJΔFuaA	*S. maltophilia* KJ *fuaA* deletion mutant; *ΔfuaA*	This study
KJΔFuaB	*S. maltophilia* KJ *fuaA* deletion mutant; *ΔfuaB*	This study
KJΔFuaC	*S. maltophilia* KJ *fuaA* deletion mutant; *ΔfuaC*	This study
KJΔABC	*S. maltophilia* KJ *fuaABC operon* deletion mutant; *ΔfuaABC*	This study
*Escherichia coli*		
DH5α	F- φ80d*lacZΔM15* Δ(*lacZYA-argF*)*U169 deoR recA1 endA1 hsdR17* (r_k_^-^ m_k_^+^) *phoA supE44λ^-^ thi-1 gyrA96 relA1*	Invitrogen
S17-1	λ pir+ mating strain	[33]
Plasmids		
pEX18Tc	*sacB oriT*, Tc^r^	[34]
pRK415	Mobilizable broad-host-range plasmid cloning vector, RK2 origin; Tc^r^	[35]
pTxylE	Plasmid containing the *xylE* gene, Amp^r^	[19]
pΔFuaR	pEX18Tc with an internal-deletion *fuaR* gene; Tc^r^	This study
pΔFuaA	pEX18Tc with an internal-deletion *fuaA* gene; Tc^r^	This study
pΔFuaB	pEX18Tc with an internal-deletion *fuaB* gene; Tc^r^	This study
pΔFuaC	pEX18Tc with an internal-deletion *fuaC* gene; Tc^r^	This study
pΔABC	pEX18Tc with an internal-deletion *fuaABC* operon; Tc^r^	This study
pFua23	pEX18Tc with a *xylE* gene inserted into the intergenic region downstream *fuaC* gene; Tc^r^	This study
pFuaA_xylE_	pRK415 with a 108-bp DNA fragment upstream from the *fuaA* start codon and a *P_fuaA_::xylE* transcriptional fusion	This study
pFuaB_xylE_	pRK415 with a 237-bp DNA fragment upstream from the *fuaB* start codon and a *P_fuaB_::xylE* transcriptional fusion	This study
Primers		
C1st	5′- TTATCGAATTCGCGCACCCAAC -3′	This study
AB-F	5′- CTTCTGGAGCTGCTGGAC-3′	This study
AB-R	5′- GCTCAGCATCGACAGCAC-3′	This study
BC-F	5′- GAGTGTGACCATCACCCC -3′	This study
BC-R	5′- CGCCATACAGTTGCCACC -3′	This study
FuaAQ-F	5′- CACCGGGATCACAGGAAC -3′	This study
FuaAQ-R	5′- CAGCAGACCGTAGAGCAG -3′	This study
FuaBQ-F	5′- GTCGCCGCACTGTCCATC -3′	This study
FuaBQ-R	5′- GCTGCTGACCGCTGCATC -3′	This study
FuaCQ-F	5′- GCAATCACACGCTCGCTG -3′	This study
FuaCQ-R	5′- TGGGCACCCTTCTGCTTC -3′	This study
rDNA-F	5′- GACCTTGCGCGATTGAATG -3′	[17]
rDNA-R	5′- CGGATCGTCGCCTTGGT -3′	[17]
1F	5′- CGCCGAATTCGCCGTGCTGACCGAAC -3′	This study
1R	5′- GCATCTAGACCTGCTCATCGCC -3′	This study
2F	5′- GTCCGGATCCAGGTCAAAGCCGGGGAG -3′	This study
2R	5′- CCCTGCAGGTGGCGAGTGTGGCG -3′	This study
3F	5′- CTGGTACCCCCGCTTACCCATC -3′	This study
3R	5′- CAGTGTCTAGACGACAGCAATC -3′	This study
4F	5′-GAAGCTTGGTACCCGCAGCCTGCGTATC -3′	This study
4R	5′- GGCAAGCTTGGATCCACGTACTGTCC -3′	This study
5F	5′- CAAAGCTTTCGCTTCCTTTGAC -3′	This study
5R	5′- TTATCGAATTCGCGCACCCAAC-3′	This study

### Construction of *fuaR*, *fuaA*, *fuaB*, *fuaC*, and *fuaABC* Mutants

**Figure 1:**
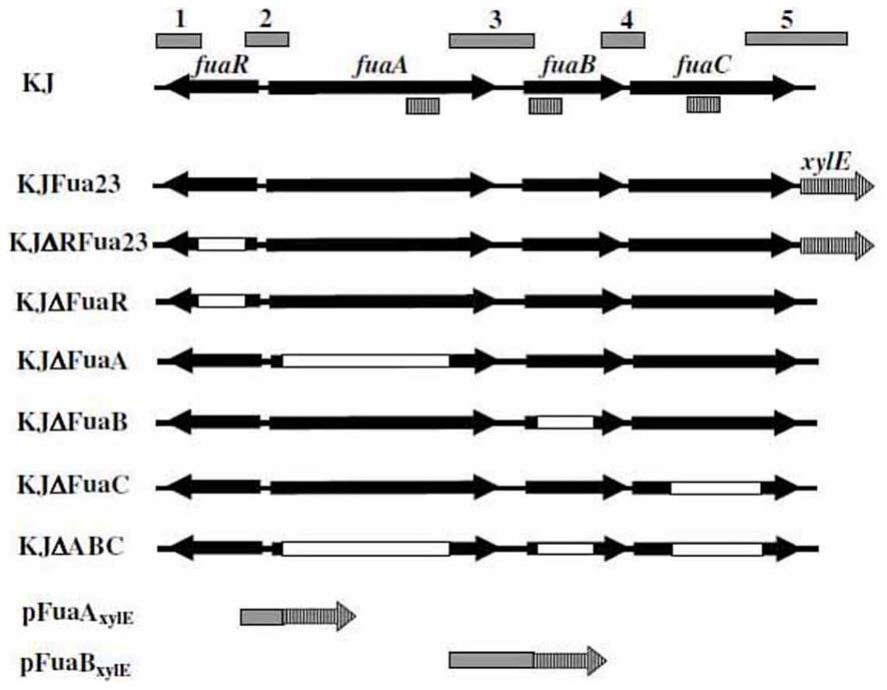
Organization of the *fuaABC* operon and *fuaR* of *S. maltophilia.* The orientation of each gene is indicated by an arrow. The approximate extent of the deletion mutants is indicated by the white bar. The gray bars (labeled as 1 to 5) indicate the PCR amplicons used for the plasmids construction. Bars with vertical lines represent the qRT-PCR amplicons. Arrows with vertical line represents the *xylE* genes.

Five PCR amplicons (labeled as 1–5 in Fig. 1) were amplified using specific primers (Table 1), to which appropriate restriction sites were added for subsequent cloning into pEX18Tc. (Primer sets 1F/1R, 2F/2R, 3F/3R, 4F/4R, and 5F/5R for amplicons 1–5, respectively). Amplicons 1 and 2 were subsequently cloned into pEX18Tc to yield the recombinant plasmid pΔFuaR, in which the cloned *fuaR* gene was partially deleted. The construction of pΔFuaA, pΔFuaB, pΔFuaC, and pΔABC followed the similar strategy, i.e., assembling the amplicons of 2 and 3, 3 and 4, 4 and 5, as well as 2 and 5, respectively. Plasmids pΔFurA, pΔFuaA, pΔFuaB, pΔFuaC, and pΔABC were mobilized to *S. maltophilia* by conjugation [17]. The deleted allele was introduced into the chromosome by a double-crossover event and the deletion mutants were selected under the presence of tetracycline (30 mg/L)/norfloxacin (2.5 mg/L) and 10% sucrose. The resultant mutants were named as KJΔFuaR, KJΔFuaA, KJΔFuaB, KJΔFuaC, and KJΔABC. The correctness of the mutants was checked by colony-PCR amplification [18] and sequencing.

### Construction of the *fuaABC-xylE* Single-copy Fusion Strain, KJFua23

A 755-bp DNA fragment (labeled as amplicon 5 in Fig. 1) containing the C-terminus of the *fuaC* gene and downstream of the *fuaC* gene was obtained by PCR using the primer sets of 5F/5R and cloned into pEX18Tc, yielding plasmid p5. A *xylE* cassette was retrieved from pTxylE [19] and inserted into the PstI site of p5, generating plasmid pFua23. The orientation of *xylE*, confirmed by sequencing, was the same as that of the *fuaABC* gene. The conjugation between *S. maltophilia* KJ and *E. coli* S17-1(pFua23) was performed as previously described [17]. The *xylE* gene in pFua23 was inserted onto the intergenic region (IG) downstream the *fuaC* gene, without disrupting any gene. This construction yielded a *fuaABC-xylE* transcription fusion in KJFua23 chromosome and the expression of the *xylE* gene can represent the expression *fuaABC* operon.

### Construction of Promoter-*xylE* Transcriptional Fusion Plasmids, pFuaA_xylE_, pFuaB_xylE_

To determine the possible promoter regions of *fuaABC* cluster, two *xylE* transcriptional fusions, pFuaA_xylE_ and pFuaB_xylE_, were constructed using the pRK415 vector. PCR amplicon 2 and 3 (Fig. 1) were cloned into pRK415 and the *xylE* gene was then inserted following the amplicon fragment to yield plasmid pFuaA_xylE_ and pFuaB_xylE_, respectively. The orientation of the *xylE* gene in constructs was opposite to that of *P_lacZ_* of the pRK415 vector. The promoter fragments assayed contained 108 bp upstream the *fuaA* gene in pFuaA_xylE_ and 237 bp upstream the *fuaB* gene in pFuaB_xylE_.

### Induction Assay

The strains tested were cultured overnight at 37°C with continuous shaking. The overnight culture was added to the fresh LB broth with an initial OD_450 nm_ of 0.2 and incubated for a further 30 min. The inducer was added as indicated. Control group without inducer was established. After incubation for a further 6 h, the cells were harvested for the catechol 2,3-dioxygenase (C23O) activity assay.

### Determination of Catechol 2,3-dioxygenase (C23O) Activity

Catechol-2,3-dioxygenase is encoded by the *xylE* gene and its activity was measured as described previously [20]. The rate of hydrolysis was calculated by using 44,000 M^−1^ cm^−1^ as the extinction coefficient. One unit of enzyme activity (Uc) was defined as the amount of enzyme that converts 1 nmole substrate per minute. The specific activity was expressed as Uc/OD_450 nm._

### Susceptibility Test

The MICs of the antimicrobials were determined with the standard agar dilution method on Mueller-Hinton agar as recommended by the CLSI [21]. After incubation of the paltes at 37°C for 18 h, the MIC was determined by obsreving the lowest concentration of the antimicrobial in which bacterial growth was inhibited. The MICs were determined alone or in combination with 20 mg/L of fusaric acid or 10 mg/L of carbonyl cyanide 3-chlorophenylhydrazone (CCCP).

### Reverse Transcriptase-PCR (RT-PCR)

Total RNA from *S. maltophilia* was isolated with a PureLinkTM Total RNA Purification System (Invirtogen, Carisbad, CA, USA) according to the manufacturer’s instruction. RNase-free DNaseI (Invitrogen, Carisbad, CA, USA) was used to eliminate DNA contamination. RT-PCR was carried out to firstly amplify the first-strand cDNA using the MMLV Reverse Transcriptase 1^st^ Strand cDNA Synthesis Kit (Epicentre Biotechnologies, Taiwan), and then PCR amplification of the cDNA was performed with *Taq* DNA polymerase. The primers used are listed in Table 1.

### Quantitative Real-Time PCR (QRT-PCR)

DNA-free RNA was prepared as aforementioned protocol. cDNA was synthesized from DNA-free RNA with a random 6-mer primer using the MMLV Reverse Transcriptase 1^st^ Strand cDNA Synthesis Kit (Epicentre Biotechnologies, Taiwan). Quantitative real-time PCR (QRT-PCR) was, then performed in the ABI Prism 7000 Sequence Detection System (Applied Biosystems) using the Smart Quant Green Master Mix (Protech Technology Enterprise Co., Ltd.) according to the manufacturer’s protocols. Relative quantities of mRNA from each gene of interest were determined by the comparative cycle threshold method [22]. The mRNA of 16S rDNA was chosen as the internal control to normalize the relative expression level. The individual target gene was amplified with the primers listed in Table 1 (FuaAQ-F/FuaAQ-R, FuaBQ-F/FuaBQ-R, FuaCQ-F/FuaCQ-R and rDNA-F/rDNA-R for *fuaA*, *fuaB*, and *fuaC* genes and 16S rDNA, respectively). Each experiment was performed at least three times.

### Bioinformatics Analysis

Multiple sequence alignments among the assayed proteins were constructed using the ClustalX program. Phylogenetic analysis was performed using *phylip package3.69*. DNA distances were calculated by *DNADist* using the Kimura model. Phylogenetic trees were constructed using the Neighbor-Joining methods. The bootstrap number was obtained in 1000 replications.

### Nucleotide Sequence Accession Number

The nucleotide sequences of *S. maltophilia* KJ *fuaR-fuaABC* cluster have been deposited in GenBank under accession no. JX524207.

## Results

### Fusaric Acid-resistance-like Gene Cluster of *S. maltophilia*

The MIC value of *S. maltophilia* KJ for fusaric acid was 512 mg/L, as established by susceptibility test (Table 2), indicating that *S. maltophilia* KJ has an intrinsic fusaric acid resistance. In an attempt to identify the fusaric acid resistance-related gene(s), a genome-wide search was performed on the *S. maltophilia* K279a genome [16]. An ORF, tagged as Smlt2796 and annotated as a putative transmembrane fusaric acid resistance efflux protein, attracted our attention. Two ORFs downstream Smlt2796, Smlt2797 and Smlt2789, had the signature sequences of periplasmic membrane fusion protein (MFP) and outer membrane protein (OMP). These features suggest that the proteins encoded by the Smlt2796-2797-2798 cluster constitute a tripartite efflux pump. A 795-bp *orf* (Smlt2795) encoding a putative AraC-type transcriptional regulator was located upstream from the Smlt2796-Smlt2797-Smlt2798 cluster and transcribed in the opposite direction, with a 70-bp Smlt2795-Smlt2796 intergenic region. This genomic organization suggests that Smlt2796-2797-2798 form an operon and Smlt2795 plays a regulator role in the expression of this operon. Therefore, the homologues of Smlt2795-2798 cluster in *S. maltophilia* KJ were named as *fuaR*, *fuaA*, *fuaB*, and *fuaC*, respectively, in this study (Fig. 1).

**Table 2:** MICs of *S. maltophilia* KJ and its derived mutants.

Strain	MIC (mg/L)													
	CHL	NAL	TET	KAM	GEM	ERY	FUA	FUA^a^	CHL^b^	NAL^b^	TET^b^	KAM^b^	GEM^b^	ERY^b^
KJ	8	8	16	256	512	64	512	128	8	8	16	128	512	64
KJΔFuaR	8	8	16	256	512	64	128	128	8	8	16	128	512	64
KJΔFuaA	8	8	16	256	512	64	128	128	8	8	16	128	512	64
KJΔFuaB	8	8	16	256	512	64	128	128	8	8	16	128	512	64
KJΔFuaC	8	8	16	256	512	64	128	128	8	8	16	128	512	64
KJΔABC	8	8	16	256	512	64	128	128	8	8	16	128	512	64

CHL, chloramphenicol; NAL, nalidix acid; TET, tetracycline; KAN, kanamycin; GEN, gentamicin; ERY, erythromycin; FUA, fusaric acid.

a Mueller-Hinton agar contains 15 mg/L CCCP in addition to antibiotic indicated.

b Mueller-Hinton agar contains 20 mg/L fusaric acid in addition to antibiotic indicated.

To assess whether the *fuaABC* cluster is indeed responsible for intrinsic fusaric acid resistance in the wild-type *S. maltophilia* KJ, a chromosomal *fuaABC* deletion mutant was constructed. The resulting mutant KJΔABC had a four-fold decrease in the MIC of fusaric acid compared with the wild-type KJ (Table 2), supporting the hypothesis that the *fuaABC* cluster contributes to fusaric acid resistance.

### *FuaA*, *fuaB*, and *fuaC* Form an Operon and the *FuaABC* Operon is Inducibly Expressed by Fusaric Acid

To evaluate the expression of *fuaABC* cluster, reverse transcriptase-PCR (RT-PCR) on the wild-type strain KJ, cultured in LB medium without fusaric acid, was performed. Primer sets FuaAQ-F/FuaAQ-R, FuaBQ-F/FuaBQ-R, and FuaCQ-F/FuaCQ-R were used for amplification of *fuaA*, *fuaB*, and *fuaC* transcripts, respectively. No significant *fuaA*, *fuaB*, and *fuaC* transcripts were detected. The MIC difference in fusaric acid between KJ and KJΔABC was demonstrated by the susceptibility test (Table 2). In some instances, the inducers to trigger the efflux pump expression are the extruded substrates of the efflux pump [23]. Based on these observations, fusaric acid is likely an inducer of expression of the *fuaABC* cluster. To test this possibility, RT-PCR was used to examine the *fuaABC* expression of strain KJ without or with the treatment of fusaric acid. Indeed, *fuaA*, *fuaB,* and *fuaC* transcripts were observed in the fusaric acid-treated *S. maltophilia* KJ. Furthermore, we attempted to verify the possibility of the *fuaABC* operon using the transcript of the fusaric acid-treated KJ strain and RT-PCR. Primer C1st was used for the first-strand cDNA synthesis and primer pairs across *fuaAB* (primers AB-F and AB-R) and *fuaBC* (primer BC-F and BC-F) were used for PCR amplification. The amplicons with expected sizes were detected (data not shown), indicating that *fuaA*, *fuaB*, and *fuaC* are transcribed as a single unit in the fusaric acid-treated KJ strain.

To further test whether the promoter of the *fuaABC* operon is inducible by fusaric acid, a 108-bp DNA fragment upstream *fuaA* gene was used to construct the *P_fuaA_*::*xylE* transcription fusion plasmid, pFuaA_xylE_. As shown in Table 3, an insignificant C23O activity was observed in KJ(pFuaA_xylE_) when cells were grown without fusaric acid. However, the C23O activity was increased approximately 22-fold with the addition of fusaric acid (Table 3). Since a 215-bp intergenic region exists between *fuaA* and *fuaB* genes (Fig. 1), we tested the possibility that *fuaB* has its own promoter. Insignificant C23O activity was detected in KJ(pFuaB_xylE_) either in the presence or absence of fusaric acid (Table 3), indicating that there is no detectable promoter activity in the 237-bp region upstream *fuaB* gene under the conditions tested. The promoter of the *fuaABC* operon is located in the intergenic region of *fuaA* and *fuaR* genes and induced by fusaric acid.

**Table 3:** Transcriptional analysis for *fuaR-fuaABC* cluster.

	C23O activity (Uc^a^/OD_450 nm_)	
	Without fusaric acid	20 mg/L fusaric acid
KJ(pFuaA_xylE_)	10±1.7	227±30
KJ(pFuaB_xylE_)	8±1.1	5±0.9
KJΔFuaR(pFuaA_xylE_)	58±6.7	9±1.4

a One unit of catechol 2,3-dioxgenase is defined as a 1 nanomole of catechol hydrolyzed per minute. Results are expressed as the mean ± SD of three independent determinations.

### Construction of a Single-copy *fuaABC*-*xylE* Transcription Fusion Strain, KJFua23

For the convenience of monitoring the *fuaABC* operon expression in following assays, a chromosomal *fuaABC* transcription fusion construct, KJFua23, was constructed by inserting a *xylE* reporter gene downstream of the *fuaC* gene without disruption of any gene (Fig. 1). The C23O activity of KJFua23 was increased more than 28-fold under fusaric acid-treated condition (4±0.8 v.s. 113±15 Uc/OD_450 nm_). To confirm this result, qRT-PCR was performed to analyze the transcripts of *fuaA*, *fuaB*, and *fuaC* genes of KJFua23 strain with or without fusaric acid. As expected, the transcripts of the three genes were increased to a similar extent in the presence of fusaric acid. Construct KJFus23 is adequate for monitoring the *fuaABC* operon expression.

### Inducibility of the *fuaABC* Operon

To investigate whether other compounds, in addition to fusaric acid, will trigger the expression of the *fuaABC* operon, the C23O activities of KJFua23 with and without the treatment of compounds were measured. The compounds tested included chloramphenicol, nalidixic acid, tetracycline, kanamycin, gentamicin, and erythromycin at a concentration of 1/4 MIC (Table 2). Among the six compounds tested, none was demonstrated to be potent inducers for the *fuaABC* expression.

To further elucidate whether the inducibility of the *fuaABC* operon is fusaric acid concentration dependent, the C23O activities of KJFua23 treated with fusaric acid of different concentrations (20, 30, 60, 90 mg/L respectively) were measured. The C23O activities of KJFua23 did not significantly change at the concentrations tested.

**Figure 2:**
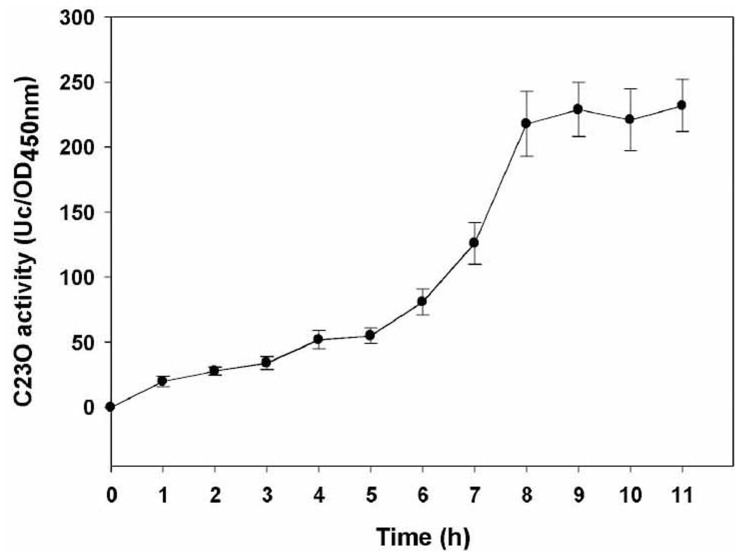
Induction of C23O activity in *S. maltophilia* KJFua23. The overnight culture of strain KJFua23 was subcultured to the fresh LB broth with an initial OD_450 nm_ of 0.2 and incubated for a further 30 min. Fusaric acid of 20 mg/L was added and the C23O activity was recorded at 1 h intervals. The error bars indicate standard deviations (n = 3).

The induction course of *fuaABC* was monitored by recording the C23O activity at 1 h intervals for strain KJFua23 after the addition of 20 mg/L fusaric acid. The C23O activity was detectable at the first sampling without any apparent lag phase and gradually increased with time. Maximum C23O activity was obtained after 8 h of induction and lasted for at least 3 h (Fig. 2).

### Role of FuaR in Expression of the *fuaABC* Operon

The *fuaABC* operon is separated by 70 bp from an upstream *fuaR* gene, an AraC-type transcriptional regulator transcribed divergently from *fuaABC*. To assess the role of *fuaR* in the *fusABC* expression, *fuaR* deletion was engineered into the wild-type KJ, yielding mutant KJΔFuaR. The promoter activities of *P_fuaABC_* in the wild-type and *ΔfuaR* background strains were assessed by determining the C23O activities of KJ(pFuaA_xylE_) and KJΔFuaR(pFuaA_xylE_). KJ(pFuaA_xylE_) had an inducible C23O activity phenotype in the presence of fusaric acid (22-fold increase), while KJΔR(pFuaAxylE) showed an increase in the basal-level expression (5-fold increase) along with a loss of inducibility (Table 3). Consequently, FuaR functions as a repressor of the *fuaABC* operon in the absence of a fusaric acid inducer and as an activator in its presence. FuaR is essential for the inducibility of the *fuaABC* operon.

The fusaric acid MIC of KJΔFuaR was evaluated. KJΔFuaR exhibited a fusaric acid MIC of 128 mg/L as low as strain KJΔABC, further supporting the hypothesis that FuaR is essential for *fuaABC* operon induction.

### Substrates Profile of the FuaABC Pump

It is well known that several tripartite pumps in Gram-negative bacteria can extrude a variety of substrates [12]. Therefore, it is of interest to decipher whether the FuaABC pump can extrude other antimicrobials, in addition to fusaric acid. However, the *fuaABC* operon is intrinsically quiescent and inducibly expressed by fusaric acid, but not by other antibiotics tested. Consequently, it is unreasonable to evaluate the substrates extruded by the FuaABC pump using the standard susceptibility test. As a result, the susceptibility test was comparatively assayed in the absence and presence of 20 mg/L fusaric acid. The wild-type KJ and KJΔABC failed to demonstrate differences in MIC value for the antimicrobials tested in the fusaric acid-addition counterpart (Table 2), indicating that the FuaABC pump cannot extrude the antimicrobials tested except fusaric acid.

### Inactivation of FuaABC Pump Components

To examine the role of each component of the FuaABC pump in fusaric acid resistance, the *fuaA*, *fuaB*, and *fuaC* genes were independently deleted, yielding deletion mutants KJΔFuaA, KJΔFuaB, and KJΔFuaC, respectively. KJΔFuaA, KJΔFuaB, and KJΔFuaC reduced the MIC of fusaric acid to the values of KJΔABC (Table 2), indicating that each component of the FuaABC pump is essential for pump function and cannot be substituted by other proteins.

### The Effect of CCCP on FuaABC Pump Activity

The proton potential of cytoplasmic membrane and ATP hydrolysis have been reported to provide energy for efflux pumps [15]. FuaA displayed ten transmembrane segments (TMS) predicted by the TMHMM tool (http://www.cbs.dtu.dk/services/TMHMM/), but did not have any nucleotide binding domain (NBD), a critical domain involved in the ATP hydrolysis. This observation suggests that the energy source for the FuaABC pump is likely the transmembrane proton gradient, and not ATP hydrolysis. To test this hypothesis, the MIC of fusaric acid was determined in the presence of the proton uncoupler carbonyl cyanide *m*-chlorophenylhydrazone (CCCP, 15 mg/L). Table 2 shows that the presence of CCCP reduced the fusaric acid resistance of the wild-strain KJ to the same level as that of KJΔABC, supporting that the CCCP inhibits the activity.

### Phylogenetic Analysis of FuaA

**Figure 3:**
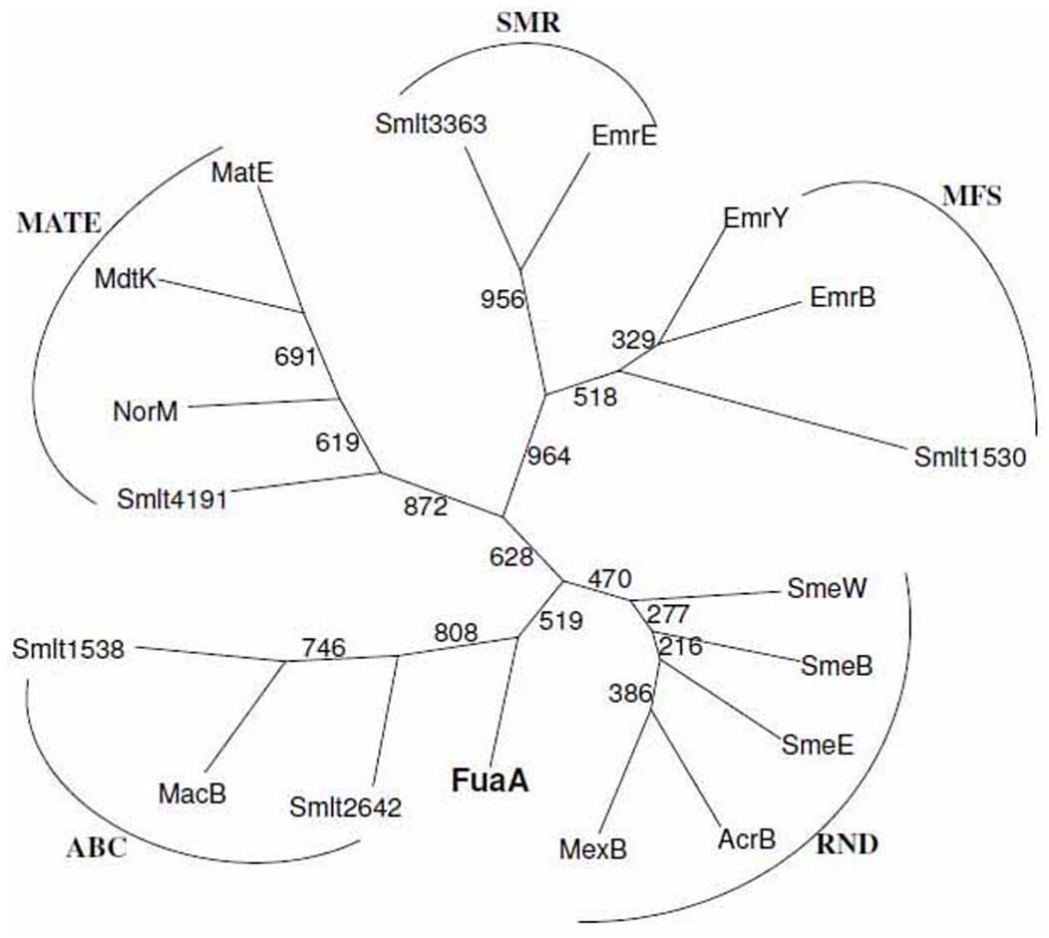
Phylogenetic analyses of FuaA protein of *S. maltophilia*. The proteins assayed in the phylogenetic tree include five RND-type transporters (AcrB of *E. coli*, MexB of *P. aeruginosa*, SmeB of *S. maltophilia*, SmeE of *S. maltophilia*, and SmeW of *S. maltophilia*), three MFS-type transporters (EmrB and EmrY of *E. coli*, and Smlt1530 of *S. maltophilia*), three ABC-type transporters (MacB of *E. coli* and Smlt1538 and Smlt2642 of *S. maltophilia*), four MATE-type transporters (NorM of *Vibro parahaemolyticus*, MdtK of *E. coli*, MatE of *Enterobacteriaceae bacterium*, and Smlt4191 of *S. maltophilia*), two SMR-type transporters (EmrE of *E. coli* and Smlt3363 of *S. maltophilia*), and FuaA of *S. maltophilia.*

The bacterial efflux pumps are classified as five families, RND, MFS, ABC, MATE, and SMR [14]. The inner membrane transporter protein FuaA investigated in this study cannot be clearly classified as a member of one of these families. A conserved domain of fusaric acid resistance protein family was identified in amino acid residues 40–180 of FuaA protein. Therefore, the phylogeny between FuaA and the known inner membrane transporters of the five efflux pump families is of interest. Five, three, three, four, and two inner membrane transporters of RND-, MFS-, ABC-, MATE-, and SMR-type of gram-negative bacteria, respectively, were selected for phylogenetic analysis. The representatives of inner membrane transporters mainly focus on *E. coli*, *P. aeruginosa*, and *S. maltophilia*, and are known to be a component of tripartite efflux pumps except MATE- and SMR-type. A phylogenetic tree was constructed from FuaA and the 17 proteins (Fig. 3). As expected, each type transporter formed its own phylogenetic cluster, labeled as RND, MFS, SMR, MATE, and ABC clusters in Fig. 3. FuaA formed a separate branch close to the ABC-type transporter cluster.

## Discussion

The classification of bacterial efflux pumps has been well described and includes the RND, MFS, ABC, MATE, and SMR families [14]. The transporter system for bacteria to adapt the environmental stress is diverse and complex. Phylogenetic analysis (Fig. 3) revealed that the FuaABC pump proposed in this article should be classified as a subfamily of ABC-type family or a new family, and its pump activity relies on the membrane proton gradient.

Although there are increasingly fusaric acid resistance genes annotated in the finished bacterial genome sequences, the actual role of these genes has been barely described. The sole actual demonstration is the *fusABCDE* operon of *Burkholderia cepacia* [5]. The FusA and FusE proteins of *B. cepacia*, which are an outer membrane protein and a membrane fusion protein, show 32% and 35% protein identity to FuaC and FuaB proteins of *S. maltophilia*, respectively. The most interesting finding is that the 142-aa FusB, 346-aa FusC, and 208-aa FusD proteins of *B. cepacia* exhibit 45%, 22%, and 39% protein identity to the N-terminus, central region, and C-terminus of the 656-aa FuaA protein of *S. maltophilia*, respectively. This suggests that the FusB, FusC, and FusD in *B. cepacia* can assemble to form an inner membrane transporter, like the FuaA in *S. maltophilia*.

The FuaR transcriptional regulator proposed in this study belongs to the AraC-type family. Most characterized proteins of the AraC family are positive transcriptional regulators [24]. However, some AraC-type regulators can function as a repressor or a positive regulator depending on the presence or absence of appropriate effectors, for example, the AraC protein from *E. coli* [25], [26], the YbtA protein from *Y. pestis* [27], and the FuaR protein from *S. maltophilia* described in this article. According to the protein length, the AraC family regulators can be distinguished into two types. The first type AraC regulator generally consists of more than 250 amino acids with an approximately 99-amino-acid helix-turn-helix (HTH) DNA-binding motif at its C terminus. The N terminus of this type AraC regulator functions as the signal receptor which can bind to the effectors. Upon the effectors binding, the AraC regulator can act as a switch with its activity being radically altered. The AraC, XylS, and Rob of *E. coli* belong to the first type AraC regulator [26], [28]. The second type AraC regulator is shorter in length, roughly 100–150 amino acids which mainly conserve the HTH motif and are devoid of the effectors binding domain. In general, the transcription of this type AraC regulator is controlled by another regulator. The SoxR and MarR of *E. coli* are representatives of the second type AraC regulator [29], [30]. The FusR proposed in this article is 264 amino acids in length and has a HTH DNA-binding motif between residues 180 and 255. Furthermore, the regulatory role of FusR toward the *fusABC* operon can switch depending on the presence or absence of fusaric acid and the inducibility of the *fuaABC* operon is FusR protein dependent (Table 3). These observations strongly support the hypothesis that the FusR of *S. maltophilia* should be classified into the first type AraC regulator and its N terminus may contain a fusaric acid-responsible domain to play a regulatory role in the *fuaABC* expression. Based on the results of this study, we propose a possible transcriptional regulation mechanism for the *fusABC* operon expression in *S. maltophilia*. In the absence of fusaric acid, FusR binds onto the *fusR-fusA* intergenic region, forming a closed configuration, which is inaccessible by RNA polymerase, repressing *fuaABC* transcription. In the presence of fusaric acid, fusaric acid binds with FusR, changing the DNA-FuaR-fusaric acid configuration into an open state that is accessible by RNA polymerase, which, in turn, induces *fuaABC* expression. It is worthily mentioned that in the absence of FuaR, fusaric acid does not function as an inducer, even represses the expression of *fuaABC* operon (Table 3, strain KJΔFuaR(pFuaA_xylE_)). Whether there is another transcriptional regulator, other than FuaR, involved in the expression of *fuaABC* operon remains to be elucidated.

A variety of *S. maltophilia* genome sequences have been released, including the clinical isolate strain K279a [16] and D457 [31] as well as plant isolate strains R553-1 and RR-10 [32]. All of these *S. maltophilia* genomes contain a repertoire of compounds resistance-associated genes to resist the environmental pressures. In this study, a fusaric acid-resistance efflux pump, *fuaABC*, which appears to be responsible for endophytic fitness has been identified in clinical isolate *S. maltophilia* KJ and its function is well conserved. Sometimes, the xenobiotics-extrusion efflux systems also extrude the antibiotics used in clinic. In this study, we assessed the clinical significance of the FuaABC pump. Results conclude that the *fuaABC* operon cannot be induced by the antibiotics tested and FuaABC pump does not function in extrusion of antibiotics tested. At this point, the FuaABC efflux pump has made little contribution to antibiotics resistance so far.
